# Carbon Felt-Based Bioelectrocatalytic Flow-Through Detectors: 2,6-Dichlorophenol Indophenol and Peroxidase Coadsorbed Carbon-Felt for Flow-Amperometric Determination of Hydrogen Peroxide

**DOI:** 10.3390/ma7021142

**Published:** 2014-02-12

**Authors:** Yue Wang, Yasushi Hasebe

**Affiliations:** 1 School of Chemical Engineering, University of Science and Technology Liaoning, 185 Qianshan Middle Road, High-tech zone, Anshan 114501, Liaoning, China; E-Mail: rosyue@163.com; 2 Department of Life Science and Green Chemistry, Faculty of Engineering, Saitama Institute of Technology, 1690 Fusaiji, Fukaya, Saitama 369-0293, Japan

**Keywords:** carbon felt, electrochemical flow-biosensor, hydrogen peroxide, 2,6-dichlorophenol indophenol, horseradish peroxidase

## Abstract

2,6-dichlorophenol indophenol (DCIP) and horseradish peroxidase (HRP) were coadsorbed on a porous carbon felt (CF) from their mixed aqueous solution under ultrasound irradiation for 5 min. The resulting DCIP and HRP-coadsorbed CF (DCIP/HRP-CF) showed an excellent bioelectrocatalytic activity for the reduction of H_2_O_2_. The coadsorption of DCIP together with HRP was essential to obtain larger bioelectrocatalytic current to H_2_O_2_. The DCIP/HRP-CF was successfully used as a working electrode unit of a bioelectrocatalytic flow-through detector for highly sensitive and continuous amperometric determination of H_2_O_2_. Under the optimized operational conditions (*i.e*., applied potential, +0.2 V *versus* Ag/AgCl; carrier pH 5.0, and carrier flow rate, 1.9 mL/min), the cathodic peak current of H_2_O_2_ linearly increased over the concentration range from 0.1 to 30 μM (the sensitivity, 0.88 μA/μM (slope of linear part); the limit of detection, 0.1 μM (S/N = 3) current noise level, 30 nA) with a sample through-put of *ca*. 40–90 samples/h.

## 1. Introduction

Porous conductive materials are useful as a working electrode unit of electrochemical flow-through detector. Compared to sensors that operate in a batch mode, sensors that operate in a flow-injection mode have many advantages: (1) potential applicability for on-line analysis (automated system); (2) high sample throughput for analysis of multiple samples; (3) negligible effect from sample dilution; and (4) a detectable concentration range and sensitivity that can be modulated by changing the sample injection volume and carrier flow rate. Up to now, reticulated vitreous carbon [1–4], microporous gold [5], and platinum mesh [6] have been used for various electrochemical flow-sensing systems.

Carbon felt (CF) is a micro-electrode ensemble of micro-carbon fiber (*ca.* 7 μm diameter) that possesses a three-dimensional random structure [7]. The CF has high conductivity and a large effective surface area, which allows large measurable current density and high electrolytic efficiency. In addition, the high porosity of CF (>90%) permits a low diffusion barrier of solution flow. Therefore, along with other electro-active porous materials, CF is useful for the electrochemical flow-through detector. Compared with other porous electrode materials, CF has the following advantages: (1) inexpensive; (2) physically and mechanically stable; (3) can be easily handled; and (4) can be easily manufactured into arbitrary shapes. Therefore, the immobilization of catalysts and biocatalysts on the CF surface has enabled the development of highly selective electrochemical flow sensors and biosensors [8–15].

The determination of H_2_O_2_ is important in chemical, biological, clinical and environmental fields. In addition, a highly functional H_2_O_2_ sensor is useful to develop biosensors for various substances by combining it with H_2_O_2_-producing oxidases. Among various analytical methods for H_2_O_2_, peroxidase-modified electrodes are useful, because highly selective, sensitive and cost-effective determination of H_2_O_2_ is possible by the peroxidase-modified electrodes. Among several peroxidase families, a horseradish peroxidase (HRP) is relatively stable, cost-effective and commercially available in highly purified form. Additionally, the structure and reaction mechanism of HRP have been well understood [16,17].

In general, most of the HRP-based H_2_O_2_-sensing electrodes detect mediated- [18–26] or direct- [27–32] reduction currents of the oxidized peroxidase-intermediates (*i.e*., compound I and II). The proposed mediator-assisted catalytic reaction schemes are as follows [18–23]:

$$\text{ferric-HRP}\;(\text{native})+H_{2}O_{2}\to \text{compound}\;I+H_{2}O$$ (1)

$$\text{compound}\;I+{\text{mediator}}_{-\text{red}}\to \text{compound II}+{\text{mediator}}_{-\text{OX}}$$ (2)

$$\text{compound}\;\text{II}+{\text{mediator}}_{-\text{red}}\to \text{ferric-HRP}\;(\text{native})+{\text{mediator}}_{-\text{OX}}$$ (3)

$${\text{mediator}}_{-\text{OX}}+n\;e^{-}\to {\text{mediator}}_{-\text{red}}(\text{at the electrod})$$ (4)

here, the reduced form of mediator (mediator_-red_) reacts with the HRP-intermediates (compound I and II) (Schemes 2 and 3), and the reduction current via mediator (Scheme 4) is monitored for amperometric detection of H_2_O_2_. Therefore, the observed catalytic current to H_2_O_2_ starts at the potential of the reduction current of the mediator (Scheme 4).

On the other hand, in some cases, the HRP-intermediates (compound I and II) can be directly reduced at the electrode surface (without mediator), as shown in the following Schemes 5 and 6:

$$\text{compound}\;I+e^{-}+H^{+}\to \text{compound II}$$ (5)

$$\text{compound II}+e^{-}+H^{+}\to \text{ferric-HRP}\;(\text{native})$$ (6)

In this case, the direct electron transfer (DET)-based the catalytic current to H_2_O_2_ start at the potential for the reduction of compound I [32].

Since electron transfer properties and the catalytic activity of immobilized enzyme are influenced by the structure, microenvironment of redox active center and interfacial properties between the enzyme and electrode, the enzyme immobilization strategy on the electrode surface would be one of the important factors which influence the sensor performance.

In previous study, we have found that coadsorption of HRP and thionine (TN, one of the phenothiazine dye families) onto a CF surface was effective to facilitate DET between the active heme center of HRP and the CF surface [10,11]. Although TN and other phenothiazine dyes are known to act as electron transfer mediators of HRP-electrodes [18–24], the relationship between the applied potential and the steady-state catalytic currents [10] and voltammetric measurement [11] revealed that the coadsorbed-TN act as promoter to facilitate DET between the HRP-intermediates and the CF. As a result, the TN/HRP-CF showed apparent DET-based catalytic current at potential range (0 to +0.4 V *versus* Ag/AgCl, at pH 7.0) [10], at which most of the TN exist as oxidized from (*E*^0^′ of TN was −0.29 V at pH 7.0), which cannot react with the HRP-intermediates. In particular, when HRP and TN were coadsorbed onto the CF surface from their mixed-aqueous solution under ultrasound irradiation [11], the DET-based catalytic current was enhanced as compared to the case without ultrasound irradiation. The specific binding interaction between TN and HRP before and/or during adsorption period may provide suitable interfacial microenvironment for a favorable orientation of HRP with the active center available for substrate and the electrode. Based on this phenomenon, we have succeeded in constructing highly sensitive electrochemical flow-through detector for H_2_O_2_ by very simple and rapid enzyme immobilization protocol.

In this paper, to expand this concept to other organic dyes (other than TN), we prepared various organic dyes [*i.e*., 2,6-dichlorophenol indophenol (DCIP); meldola’s blue (MdB); methylene green (MG)] and HRP-coadsorbed-CFs, and the cathodic peak current responses to H_2_O_2_ obtained by organic dyes/HRP-CF-based flow-through detector was compared. Among them, the DCIP/HRP-CF-based system showed the largest peak current response. Bioelectrocatalytic reaction of the DCIP/HRP-CF was evaluated by cyclic voltammetry. Operational conditions for flow amperometric determination of H_2_O_2_ (*i.e*., applied potential, carrier pH, and carrier flow rate) were optimized, and analytical performance of the developed DCIP/HRP-CF-based flow-through H_2_O_2_ detector was characterized.

## 2. Results and Discussion

### 2.1. Comparison of Various Organic Dyes

**Figure 1.**
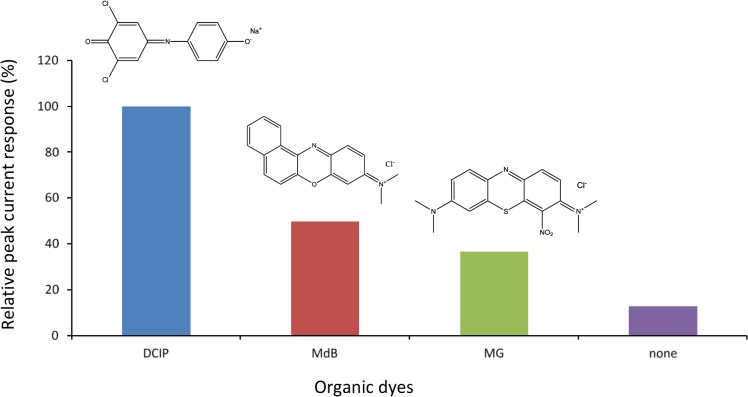
Comparison of the magnitude of cathodic peak current responses to 30 μM H_2_O_2_ obtained by various organic-dyes/HRP-CFs-based flow-through H_2_O_2_ detectors. DCIP: 2,6-dichlorophenol-indophenol; MdB: meldola’s blue; MG, methylene green. Applied potential is 0 V *vs*. Ag/AgCl. 0.1 M phosphate buffer (pH 7.0) was used as a carrier at 1.5 mL/min.

Figure 1 shows comparison of cathodic peak current responses to H_2_O_2_ obtained by various dyes/HRP-CF-based electrochemical flow-through detectors under the applied potential of 0 V *versus* Ag/AgCl at pH 7.0. The MG [22,23], MdB [26] and DCIP [33,34] are all redox-active organic dyes, and have been successfully used as electron transfer mediators of various enzyme-modified electrodes [22,23,26,33,34]. Compared to HRP-CF (without dye), all dyes/HRP-CFs-based systems showed larger cathodic peak current response to H_2_O_2_. Among them, the DCIP/HRP-CF-based system showed the largest responses.

As described in introduction section, we have found that coadsorption of TN and HRP onto CF surface was effective to facilitate DET between the active heme center of HRP and CF surface [10,11]. Therefore, taking into account role of TN as a promoter for DET, the observed organic dyes-induced signal enhancement effects (Figure 1) would be arising from two possibilities: First one is that these organic dyes act as electron transfer mediators, like previous reports [18–26,33,34]. Another one is that these organic dyes act as promoters to facilitate DET between HRP and CF, similar to the case of TN/HRP-CF [10,11].

Based on these assumptions, the differences in the magnitude of the peak current responses by various dyes/HRP-CFs systems (Figure 1) would be attributed to the differences in: (1) electrochemical properties of dyes (e.g., *E*^0^′ and *k*); (2) interaction between dyes and HRP; and (3) interaction between dyes and CF surface. Since both MG and TN belong to phenothiazine dyes and the *E*^0^′ values of MG and TN are almost same [18,19,23,24], it is reasonable to assume that MG promotes DET like TN. Therefore, smaller response of the MG/HRP-CF-based system as compared to the TN/HRP-CF system [10,11] (data not shown) may be attributed to the differences in the molecular structure of MG and TN. MG possesses more bulky two dimethyl amino group and NO_2_ group. In contrast, TN possesses smaller two amino groups. These structural differences would result in: (1) different binding interaction with HRP and (2) different interfacial properties of the adsorbed HRP (*i.e*., structure, orientation and conformation). From view point to develop highly sensitive flow-sensing system, the DCIP/HRP-CF was selected for subsequent study, and the bioelectrocatalytic reaction of the DCIP/HRP-CF for the H_2_O_2_ reduction was evaluated by cyclic voltammetry.

### 2.2. Bioelectrocatalytic Reduction of H_2_O_2_ by DCIP/HRP-CF

**Figure 2.**
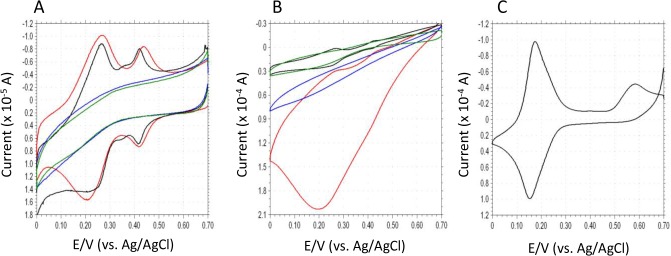
(**A**) Cyclic voltammograms (CVs) of DCIP/horseradish peroxidase-carbon felt (HRP-CF) (red line), HRP-CF (blue line), DCIP-CF (black line) and bare-CF (green line) in deoxygenated 0.1 M phosphate buffer (pH 5.0); (**B**) CVs of DCIP/HRP-CF (red line), HRP-CF (blue line), DCIP-CF (black line) and bare-CF (green line) in deoxygenated 0.1 M phosphate buffer (pH 5.0) in the presence of 0.3 mM H_2_O_2_; (**C**) CV of bare-CF in 0.2 mM DCIP-containing deoxygenated 0.1 M phosphate buffer (pH 5.0). Potential scan rate is 0.01 V/s. The starting potential is +0.7 V *vs*. Ag/AgCl.

Cyclic voltammetry is useful to verify the electron transfer properties and electrocatalytic mechanism of surface-modified electrodes. Figure 2A shows the cyclic voltammograms (CVs) of DCIP/HRP-CF, HRP-CF, DCIP-CF and bare-CF in deoxygenated 0.1 M phosphate buffer (pH 5.0). Both DCIP/HRP-CF and DCIP-CF showed two redox waves at *ca.* +0.24 V and *ca*. +0.42 V, respectively. Since the CV of free DCIP in buffer showed only one redox couple at *ca.* +0.165 V (Figure 2C), the DCIP molecule adsorbed on the CF may have two different adsorption states. As seen in Figure 2B, in the presence of H_2_O_2_, the DCIP/HRP-CF showed large cathodic current in the potential range between +0.6 V and 0 V, suggesting that the DCIP/HRP-CF exhibited an excellent bioelectrocatalytic activity for the electrochemical reduction of H_2_O_2_. Although HRP-CF showed cathodic currents in the same potential region, the observed DET-based catalytic current of HRP-CF was much smaller than the catalytic current of DCIP/HRP-CF. In contrast, DCIP-CF and bare-CF showed similar CV curves in the presence of H_2_O_2_, suggesting that the adsorbed DCIP does not have an electrocatalytic activity for the H_2_O_2_ reduction. Based on these results, it is clear that coadsorption of DCIP and HRP is effective to enhance the bioelectocatalytic current for the reduction of H_2_O_2_.

Differing from TN (*E*^0^′ = *ca.* −0.2 V), the *E*^0^′ value of adsorbed DCIP is much more positive (*E*^0^′_1_ = +0.24 V and *E*^0^′_2_ = +0.42 V), and can be seen in red line Figure 2A,B, the reduction currents of DCIP seem to increase in the presence of H_2_O_2_. Therefore, it would be reasonable to consider that the observed large catalytic currents are attributed to the DCIP-mediated reaction (Schemes 1–4). However, we cannot deny another possibility that the DCIP promotes the DET, because the shapes of CV curves of HRP-CF and DCIP/HRP-CF are both quite similar to that of previously reported HRP-adsorbed spectrographic graphite electrode, in which the DET-based response currents to H_2_O_2_ start at +0.6 V *versus* SCE (pH 6.0) [32]. In general, if catalytic cycle of mediator/HRP-system is so fast, the oxidation peak of mediator tends to decrease and/or disappear [18–21]. However, in the present DCIP/HRP-CF-based system, two oxidation peaks of DCIP are still remaining even in the presence of H_2_O_2_ (see Figure 2B, red line).

If the signal enhancement effect by DCIP is arising from the promotion of DET, the coadsorption of DCIP and HRP from their mixed solution may provide suitable interfacial microenvironment for a favorable orientation and structure of adsorbed HRP. To verify this assumption, further studies are required: e.g., (1) the observation of surface morphology of adsorbed enzyme by scanning probe microscopy; (2) analysis of the orientation of enzyme by Surface-enhanced Raman spectroscopy; (3) analysis of the binding interaction between organic dye and enzyme by spectrophotometry. These are important research topic in near future.

### 2.3. Optimization of Operational Conditions of DCIP/HRP-CF-Based Flow-through H_2_O_2_ Detector

**Figure 3.**
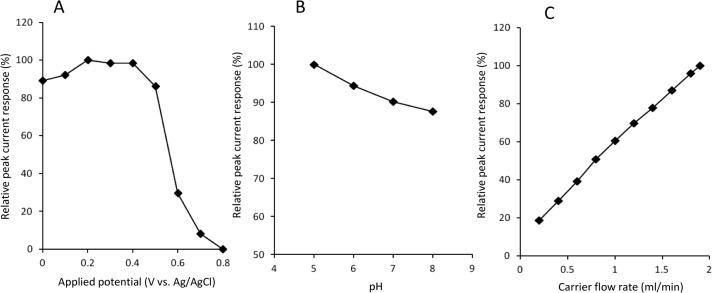
(**A**) Effect of applied potential on the relative peak current responses to 10 μM H_2_O_2_. 0.1 M phosphate buffer (pH 5.0) is used as carrier at flow rate of 1.5 mL/min; (**B**) Effect of carrier pH on the relative peak current responses to 10 μM H_2_O_2_. Applied potential is +0.2 V *vs*. Ag/AgCl. 0.1 M phosphate buffer is used as a carrier at flow rate of 1.5 mL/min; (**C**) Effect of carrier flow rate on the relative peak current responses to 10 μM H_2_O_2_. Applied potential is +0.2 V *vs*. Ag/AgCl. 0.1 M phosphate buffer (pH 5) is used as a carrier. The average values for three measurements are plotted.

Biocatalyst-modified CF is useful as a working electrode unit of CF-based electrochemical flow-bio-sensing systems [8–15]. To obtain higher peak current responses, the operational conditions (*i.e*., applied potential, carrier pH and carrier flow rate) were optimized. Figure 3A shows the effect of applied potential on the cathodic peak currents to H_2_O_2_. Apparent cathodic peak appeared at +0.7 V, and increased with changing the potential from +0.7 to +0.4 V. The peak currents seemed to reach plateau at +0.4~+0.2 V. Slight decrease in peak current in more negative potential region (from 0.1 V to 0 V) would be attributed to increased background current, which is probably due to the electrochemical reduction of dissolved oxygen in carrier. This result is essentially consistent with the CV curve of DCIP/HRP-CF obtained in the presence of H_2_O_2_ (Figure 2A). Based on this result, +0.2 V was selected as a working potential for the subsequent study.

Because pH influences the structure and activity of peroxidase-electrodes, we next studied the effect of carrier pH on the cathodic peak currents to H_2_O_2_ in the pH range from 5.0 to 8.0 (Figure 3B). The pH was adjusted by using 0.1 M phosphate buffer. Although the capacity of the buffer is weak at acidic region (pH 5.0), we confirmed that the pH was not changed during the experiments. As shown in Figure 3B, the peak current response increased with decreasing pH, and the maximum response was obtained at pH 5.0. This pH dependency is probably due to the influence on the structure of adsorbed HRP and the electrochemical reduction properties of the HRP intermediates (compound I and II) which require H^+^ addition. Based on this result, we selected pH 5.0 buffer as a carrier solution.

Carrier flow rate influences the analytical performance of the FIA system. Therefore, the effect of carrier flow rate was investigated over the range of 0.2 to 1.9 mL/min (1.9 mL/min is maximum limit of the pump used in this study). As shown in Figure 3C, the magnitude of cathodic peak current increased with increasing carrier flow rate, and the largest peak current was obtained at 1.9 mL/min. This result suggests that HRP-catalyzed H_2_O_2_ reduction on the DCIP/HRP-CF is sufficiently fast and that mass transport would be the rate determining step of this system.

### 2.4. Analytical Performance of HRP/DCIP-CF-Based Flow-through H_2_O_2_ Detector

**Figure 4.**
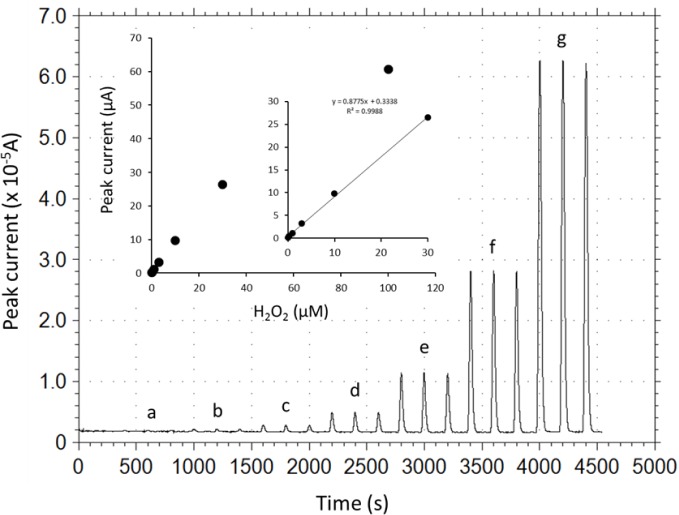
Typical cathodic peak current responses to various concentrations of H_2_O_2_ standard solutions. a, 0.1; b, 0.3; c, 1; d, 3; e, 10; f, 30; and g, 100 μM. Inset graph is calibration curve of H_2_O_2_ obtained by the DCIP/HRP-CF-based flow-through H_2_O_2_ detector. The average values for three measurements are plotted. Applied potential is +0.2 V *vs*. Ag/AgCl. 0.1 M phosphate buffer (pH 5.0) is used as a carrier at flow rate of 1.9 mL/min.

Under the optimized operational conditions (applied potential, +0.2 V; carrier pH 5.0; carrier flow rate, 1.9 mL/min), analytical performance of the DCIP/HRP-CF-based flow-through H_2_O_2_ detector was evaluated. Figure 4 illustrates typical peak current responses to various concentrations of H_2_O_2_ standard solutions, and a calibration curve for H_2_O_2_ obtained by the DCIP/HRP-CF-based flow-through detector. As can be seen, the reproducibility of the response to the same concentrations of samples seems to be good. The relative standard deviations (RSD, *n* = 3) were less than 3%. The peak width was in the range of *ca.* 40–90 s. Therefore, the sample throughput was in the range of *ca*. 40–90 samples/h.

As shown in inset graph in Figure 4, the calibration curve was linear in the H_2_O_2_ concentration range from 0.1 to 30 μM with the sensitivity of 0.88 μA/μM (correlation coefficient, 0.9988). The limit of detection was estimated to be *ca.* 0.1 μM with a signal to noise ratio of three (noise level 30 nA). Based on the electrochemical Lineweaver-Burk double reciprocal plot, the apparent Michaelis-Menten constant, *K*_m_^app^ and *I*_max_ were estimated to be 96.8 μM and 106.3 μA, respectively, based on the equation *Y* = 0.91 *X* + 0.0094 (*r*^2^ = 0.9999). The measurement reproducibility (operational stability) was ascertained by consecutive injections of 10 μM H_2_O_2_ standard solution. Repetitive 30 sample injections of 10 μM H_2_O_2_ induced no serious current decrease, and the RSD (*n* = 30) was 4.2%. Therefore, it can be safe to conclude that serious desorption of HRP and DCIP and deactivation of adsorbed HRP is negligible at least during the continuous measurement period (several hours).

To evaluate the intra-day precision (between-lot variation) of DCIP/HRP-CF, three DCIP/HRP-CFs were prepared in the same manner in the same day, and the peak current response to 30 μM H_2_O_2_ (seven consecutive injections) was measured. The RSD value (*n* = 3) for the peak current response to H_2_O_2_ obtained by the different DCIP/HRP-CF was less than 4%. This result indicates that the present adsorption protocol for the DCIP/HRP-CF has acceptable reliability even though the preparation protocol is quite easy and simple (just physical adsorption for 5 min under ultrasound irradiation), which is one of the notable advantages of this system.

Finally, storage stability was checked. When not in use, the DCIP/HRP-CF was stored in 0.1 M phosphate buffer (pH 5.0) at 4 °C in the refrigerator. Unfortunately, the DCIP/HRP-CF showed *ca.* 78% of its original activity after three days storage, and *ca.* 52% of activity after 7 days storage. Since HRP strongly adsorbs on the CF surface for long storage period [10–12,14], the lesser storage stability of the present DCIP/HRP-CF would be due to desorption of DCIP from the CF surface and/or gradual conformational change of the adsorbed HRP molecule. In fact, area of CV redox peaks of the adsorbed DCIP decreased *ca.* 60% of initial values after the 7 days storage. Furthermore, the surface resistance (*R*_ct_), which was evaluated by a Nyquist-plot of electrochemical impedance spectroscopy, increased about 1.7 times after 7 days of storage (data not shown). These results suggest that leaching of DCIP and the structural change (unfolding or spreading) of the adsorbed HRP may be the primary reason for this decrease in the activity during the storage period in buffer. Lesser storage stability of this DCIP/HRP-CF-based system may be attributed to the differences in: (1) the affinity of organic dyes on the CF; (2) strength of binding interaction with HRP; and (3) adsorption state of HRP on the CF. To improve the lesser storage stability, the following approaches would be useful: (1) film coating on the adsorbed enzyme layer (to prevent leaching of DCIP); (2) appropriate additives (e.g., conformational tightening reagent); and (3) chemical modification of HRP on the CF surface.

Table 1 summarizes the comparison of analytical performances of the present DCIP/HRP-CF-based flow-through H_2_O_2_ detector and previously reported TN/HRP-CF-based system [11]. Although the applied potential and carrier flow rate are different, the sensitivity, lower detection limit and storage stability of the present DCIP/HRP-CF-based system are unfortunately inferior to those of the TN/HRP-CF-based system [11]. However, wide linear range of the present DCIP/HRP-CF-based system would be advantage for future practical applications: e.g., analysis of H_2_O_2_ in real samples such as swimming pool water [35], and analysis of various oxidase substrates by the combination with various oxidases [13,14,36–38].

## 3. Experimental Section

### 3.1. Reagents and Materials

A carbon felt (CF) sheet (GF-20-3F, which was prepared by pyrolysis of polyacrylonitrile at 2000°C, density 0.13 g/cm^3^) was obtained from Nippon Carbon Ltd. (Tokyo, Japan). Horseradish peroxidase (HRP; EC 1.11.1.7.; 100 units/mg), 2,6-dichlorophenol-indophenol (DCIP), methylene green (MG) and 30% hydrogen peroxide (H_2_O_2_) were purchased from Wako Pure Chemical Industry Ltd., (Osaka, Japan) and were used as received. Meldola’s blue (MdB) was purchased from Dojindo Co. (Kumamoto, Japan). All of the other chemicals were of the highest grade available, and used without further purifications. 0.1 M phosphate buffer (prepared by using KH_2_PO_4_ and K_2_HPO_4_) was used as the standard sample and carrier solutions. A standard solution of H_2_O_2_ was prepared by the dilution of 30% H_2_O_2_ with a buffer. Doubly distilled deionized water (Yokozawa Chemical Co., Ueda, Japan) was used for the preparation of all solutions throughout the experiments.

### 3.2. Preparation of Organic Dyes and HRP-Coadsorbed-CF

The CF sheet was cut into appropriate size (*i.e*., 10 mm × 3 mm × 3 mm; weight is *ca.* 12 mg; apparent volume is 90 mm^3^), and was washed in pure water under ultra-sonication for 10 min. Then the CF was immersed into organic dye and HRP-mixed aqueous solution ([organic dye] = 0.5 mM; [HRP] = 0.033 mg/mL, volume is 2.0 mL) at 25 °C for 10 min under ultrasound irradiation (40 kHz, 55 W, ultra-sound bath, AZONE, Model US-1R, Osaka, Japan). To remove weakly adsorbed species, the organic dye/HRP-CF was placed in an electrochemical flow-through cell, and the buffer was flowed at 1.9 mL/min for 1000 s with a double-plunger pump (SNK DX2000, Sanuki Industry Ltd., Tokyo, Japan).

### 3.3. Electrochemical Measurements

Cyclic voltammetry (CV) was carried out to evaluate the bioelectrocatalytic reaction of organic dye/HRP-CF with electrochemical analyzer (ALS Model 611B, BAS, West Lafayette, IN, USA) A one-compartment electrochemical glass cell was used. The dye/HRP-CF with platinum lead wire (0.5 mm diameter, 70 mm length) was used as a working electrode. A platinum wire (1 mm diameter, 50 mm length) and a Ag/AgCl electrode (BAS, ER-1B, 3M NaCl, BAS Co., West Lafayette, IN, USA) were used as an auxiliary and reference electrodes, respectively. Deoxygenated buffer was used as an electrolyte, and a N_2_ atmosphere was maintained over the solution during the measurements. Deoxygenated buffer was prepared by introducing pure N_2_ gas into buffer for at least 20 min. To obtain the interfacial properties of DCIP/HRP-CF surface, electrochemical impedance spectroscopy (EIS) was carried out with an electrochemical analyzer (ALS Model 6122A, ALS Co., Tokyo, Japan) at room temperature with a conventional one-compartment three electrode system similar to the CV measurements. A deoxygenated 0.1 M phosphate buffer (pH 5.0, 15 mL) containing 0.25 mM hydroquinone was used as an electrolyte. The applied potential was set at +0.2 V *versus* Ag/AgCl (the formal potential of the hydroquinone/*p*-quinone redox system at pH 5.0). The frequency ranged from 0.01 Hz to 10 kHz.

### 3.4. Flow Amperometric Measurements

Flow amperometric measurements were carried out with the CF-based electrochemical flow-injection analysis (FIA) system, which is essentially the same system that has been reported previously by us [8–15]. The system is composed of a double-plunger pump (SNK DX2000, Sanuki Industry Ltd., Tokyo, Japan) with a six way injection valve with 200 μL injection loop, and the dye/HRP-CF-based electrochemical flow-through detector connected to the electrochemical analyzer (ALS Model 611B, ALS Co., Tokyo, Japan). All electrochemical measurements were carried out at ambient temperature. Air-saturated 0.1 M phosphate buffer was used as a carrier. Prior to the measurements, the carrier was flowed at 1.9 mL/min for 1000 s under the working applied potential to obtain stable background currents. After the background current had reached the steady-state value, the samples were injected through a syringe filter unit (0.45 μm pore size, Dismic 3cp, Advantec, Tokyo, Japan) at regular time intervals, and the peak-shaped current, based on the HRP-catalyzed reduction of H_2_O_2_, was recorded.

## 4. Conclusions

In this study, DCIP and HRP were coadsorbed onto the CF surface from their mixed aqueous solution under ultrasound irradiation for 5 min, to construct electrochemical flow-through detector for amperometric determination of H_2_O_2_. Coadsorbed DCIP was essential to enhance the HRP-catalyzed bioelectrocatalytic reduction current of H_2_O_2_. Preparation protocol of the DCIP/HEP-CF is very simple, and the developed DCIP/HRP-CF-based electrochemical flow-through H_2_O_2_ detector enabled the continuous amperometric determination of H_2_O_2_ over the concentration range from 0.1 μM to 30 μM. In future work, the DCIP-HRP-CF can be useful to develop flow biosensing system for various oxidase substrates by coupling it with corresponding H_2_O_2_-producing oxidases (glucose [13,36], uric acid [14], lactate [36], glutamate [37], amines [38] and alcohol [39], *etc*.).

## Figures and Tables

**Table 1.** Comparison of analytical performances of the thionine (TN)/HRP-CF- and DCIP/HRP-CF-based flow-through H_2_O_2_ detector.

Sensors	Applied potential	Carrier flow rate	Linear range	Sensitivity	Detection limit	Storage stability^(1)^	Refs
	(V *vs*. Ag/AgCl)	(mL/min)	(μM)	(μA/μM)	(μM)		
TN/HRP-CF	0	3.25	0.02 to 3	4.72	0.02	85% (after 6 days)	[11]
DCIP/HRP-CF	+0.2	1.9	0.1 to 30	0.88	0.1	52% (after 7 days)	This work

(1) TN/HRP-CF and DCIP/HRP-CF were stored in 0.1 M phosphate buffers pH 7.0 and pH 5.0, respectively.
